# Prevalence, patterns, drivers, and perceived benefits of herbal medicine use in COVID-19 patients in Qatar

**DOI:** 10.1080/20523211.2025.2533258

**Published:** 2025-07-23

**Authors:** Raneem Alsheikh, Fatima R. Alsharif, Nouran Alwisi, Zachariah Nazar, Mohamed Ahmed Syed, Hamda Abdulla Qotba, Layla Al-Mansoori, Zumin Shi, Abdullah A. Shaito

**Affiliations:** a College of Medicine, QU Health, Qatar University, Doha, Qatar; b College of Pharmacy, QU Health, Qatar University, Doha, Qatar; c Department of Clinical Research, Primary Health Care Corporation (PHCC), Doha, Qatar; d Biomedical Research Center, QU Health, Qatar University, Doha, Qatar; e Department of Biomedical Sciences, College of Health Sciences, QU Health, Qatar University, Doha, Qatar; f Department of Human Nutrition, College of Health Sciences, QU Health, Qatar University, Doha, Qatar

**Keywords:** COVID-19, herbal medicine, CAM, self-prescription, Qatar, ginger, turmeric

## Abstract

**Background:**

Coronavirus disease 2019 (COVID-19), caused by the severe acute respiratory syndrome coronavirus 2 (SARS-CoV-2), emerged as a global health crisis in early 2020, leading to widespread morbidity and mortality. In Qatar, as of December 2024, the disease burden has reached over 500,000 cases and more than 600 deaths. While conventional treatments have evolved throughout the pandemic, the use of Complementary and Alternative Medicine (CAM), particularly herbal medicine, has also become prevalent. This study investigated the prevalence, reasons, uses, types, self-reported benefits, and sociodemographic determinants of utilising herbal medicine among COVID-19 patients in Qatar.

**Methods:**

A cross-sectional study was conducted among patients attending Qatar Primary Health Care Corporation (PHCC) clinics. Of the 10,000 SMS invitations that were sent, 882 survey responses were received from patients diagnosed with COVID-19 between 1 March 2020, and 30th April 2022. Following the exclusion of 31 participants due to missing data, sociodemographic data from 851 participants were analysed using logistic regression to assess predictors of herbal medicine use. Prevalence, patterns, types, and self-reported benefits were analysed using descriptive statistics.

**Results:**

Of the 851 respondents included in the analysis, 440 (51.7%) reported herbal medicine use. Herbal medicine use was associated with better-perceived health outcomes. Women were more likely to use herbal medicine than men (OR = 1.90, 95%CI: 1.30–2.77, *p* = 0.001). The most used herbal remedies were ginger (n = 347), turmeric (n = 207), and garlic (n = 155). Family tradition (42%), the belief that herbs are natural (34.8%), and the desire to improve health and survival (31.1%) were the leading drivers of herbal medicine use.

**Conclusion:**

Herbal medicine use was prevalent (51.7%) during COVID-19 in Qatar, with a higher prevalence of use among women. This prevalence was driven by cultural beliefs and perceived health benefits. The study contributes insights to guide future research, policy, and practice toward safe and evidence-informed integration of herbal medicine in pandemic preparedness and broader healthcare strategies.

## Background

Coronavirus disease 2019 (COVID-19), caused by the severe acute respiratory syndrome coronavirus 2 (SARS-CoV-2), emerged as a public health challenge in early 2020. On 11th March 2020, the World Health Organization (WHO) officially declared the COVID-19 outbreak a pandemic. The disease is primarily characterised by severe acute respiratory illness, with symptoms such as fever, cough, and dyspnoea (Cascella et al., 2024). In severe to critical illness, patients frequently develop acute respiratory distress syndrome (ARDS), a life-threatening condition requiring hospitalisation. Extrapulmonary manifestations of the disease include gastrointestinal symptoms, myocardial injury, and acute kidney failure (Cascella et al., 2024; Vetter et al., 2020).

The pandemic had significant morbidity and mortality tolls. As of December 2024, approximately 777 million cases of COVID-19 were reported globally, with an associated mortality of 7 million individuals (WHO, 2025). In Qatar, the total burden amounted to over 500,000 cases and more than 600 deaths (WHO, 2025).

Early therapeutic options for COVID-19 included hydroxychloroquine, initially considered due to its proposed antiviral properties (Prodromos & Rumschlag, 2020). However, clinical trials demonstrated its limited efficacy, and concerns about its side effects led to decreased use (Chivese et al., 2021). The mainstay of treatment since has depended on the severity of the disease. Patients with no risk factors for disease progression are treated symptomatically (Lui & Guaraldi, 2023). Currently, patients with mild to moderate disease, whether outpatient or hospitalised, are treated with antiviral agents or monoclonal antibodies (Lui & Guaraldi, 2023). Hospitalised patients with severe or critical illness are treated with the antiviral drug remdesivir and corticosteroids, with the potential combination of immunomodulatory agents such as JAK inhibitors or IL-6 inhibitors in the case of rapid deterioration (Lui & Guaraldi, 2023).

Despite the development of vaccines that have proven highly efficacious in reducing transmission and alleviating symptoms in infected patients, COVID-19 has not been eradicated (Graña et al., 2022; Roknuzzaman et al., 2024). SARS-CoV-2 is a rapidly mutating virus, with new variants that emerge and are unaffected by existing vaccines (Zhao et al., 2024). Thus, even vaccinated individuals are at risk of contracting the disease, particularly from new variants of the virus (Zhao et al., 2024). Additionally, many patients experience persistent, long-term complications following infection, a condition now recognised as ‘Long COVID’ (Koc et al., 2022). This syndrome can include a wide range of symptoms, such as fatigue, cognitive dysfunction, and respiratory symptoms, significantly impacting quality of life (Davis et al., 2021). As current preventive measures do not provide complete protection against infection or post-infection complications, the need for therapeutic options persists (Robinson et al., 2022). In the absence of definitive therapies, individuals increasingly sought complementary and alternative medicine (CAM) as a means of therapeutic support (Chowdhuri & Kundu, 2020).

CAM use surged during the COVID-19 pandemic, with a systematic review of 62 studies estimating a global prevalence of approximately 64% (Kim et al., 2022). This widespread use was influenced by various factors, including disease burden, previous CAM use, and perceived benefits for immunity and symptom relief (Chowdhuri & Kundu, 2020). Particularly, herbal medicine, a key CAM modality, garnered popularity during the pandemic as individuals sought alternative remedies to enhance immunity, alleviate symptoms, or prevent infection (Liu et al., 2022). Interestingly, ongoing clinical trials are testing herbal products as alternative medicines to treat COVID-19 (Jaber et al., 2022). Notably, COVID-19 lockdowns and the increased online medicine procurement also drove self-medication behaviours, further encouraging unsupervised herbal medicine use (Jairoun et al., 2021; Kazemioula et al., 2022; Yalçin et al., 2022). Among the most used herbs were ginger, garlic, turmeric, and green tea (Pieroni et al., 2020). These herbs were favoured for their well-recognised anti-inflammatory, antioxidant, and immune-boosting properties (Singh et al., 2021). In addition, using herbal medicine as a complementary therapy to conventional drugs has shown promising results, with meta-analyses reporting improved effectiveness, enhanced symptom relief, and reduced progression to severe COVID-19 (Ang et al., 2022b; Du et al., 2021).

In Qatar, herbal medicine remains an integral part of traditional Arabic and Islamic healing practice (Alrawi et al., 2017). Around 40% of middle-aged women report using CAM, with herbal and nutritional therapies being the most common (Gerber et al., 2014). CAM is also frequently used in chronic disease management; for instance, 53% of patients with type 2 diabetes reported CAM use, primarily in the form of herbal powders (Mohamed et al., 2016). Furthermore, 83% of general practitioners in Qatar support the integration of CAM into healthcare (Al Shaar et al., 2010).

To our knowledge, despite the widespread use of herbal medicine during the COVID-19 pandemic, no studies have investigated its use among patients in Qatar. Given Qatar’s highly diverse population, studying this group allows for the exploration of trends that may not be evident in studies conducted in more homogenous populations. We hypothesise that herbal medicine use varies by sociodemographic factors and perceived benefits. Therefore, this study aimed to investigate the prevalence, reasons, uses, types, self-reported benefits, and sociodemographic determinants of herbal medicine use among COVID-19 patients in Qatar.

## Methods

### Study design and participants

A cross-sectional study was conducted to investigate the use of CAM among COVID-19 patients in the State of Qatar. The study proposal, consent form, and survey questionnaire were approved by the Primary Healthcare Corporation (PHCC) Institutional Review Board (IRB) with reference number PHCC/DCR/2022/06/040 and Qatar University IRB committee approval number QU-IRB 1779-E/22.

Participants were considered eligible if they were registered at one of the 26 PHCC clinics, were male or female adults aged ≥18 years, were conversant in English or Arabic, and were previously diagnosed with COVID between 1 March 2020, and 30 April 2022. A total of 10,000 invitations were sent via SMS to participate in an online survey developed with Google Forms. Out of the 882 responses received, 31 participants were excluded due to missing data on herbal medicine use (yes, no).

#### Sample size

For sample size calculation, we applied Cochran's formula for proportions using categorical data $\left(n=\frac{z^{2}p\cdot q}{d^{2}}\right)$, yielding a required sample size of 601 participants.

Where n is the sample size, Z = 1.96 for 95% confidence, *p* = 0.5, *q* = 1−*p* = 0.5, and *d* is the acceptable margin of error =   0.04.

This study included 851 patients with a confirmed history of COVID-19.

### Survey instrument

The questionnaire was informed by the relevant literature. Questions were included to collect patient characteristics and symptoms during the COVID-19 pandemic and herbal medicines use. The questionnaire was first created in English and then translated into Arabic. The Arabic version was back-translated to English to confirm the questionnaire's parallel-form reliability. Both the Arabic and English versions of the questionnaire were pilot-tested with a convenient sample of 32 individuals to assess clarity, simplicity, and logical structure. Cronbach’s alpha was used to test internal consistency, yielding an alpha level of 0.8, signifying that the survey items were reliable and internally consistent. The final English and Arabic versions were refined by rewording certain questions and removing any redundancy. The final questionnaire (see Supplemental Material) consisted of 25 questions and took approximately 6–8 min to complete. It was divided into 4 sections: (A) sociodemographic characteristics, (B) information about the participant’s general health, (C) participant’s COVID-19 symptoms and characteristics, (D) herbal medicine use, types and patterns of use, and (E) self-reported benefits and harms of the herbal medicines used. The respondents could only reply once to the survey and no incentives were offered.

### Covariates

Sociodemographic variables that were used in the primary outcome analysis included age (18–34, 35–54, 55+), sex, nationality (Qatari, non-Qatari), education level (below university, university or above), marital status (married, divorced/widowed, never married), and employment status (no, yes). COVID-19 severity levels were divided, according to cut-offs in a data-driven approach, into mild, moderate, or severe. Patients were classified as having a severe disease if they required hospitalisation for any reported symptoms. Their disease was considered moderate if they reported five or more symptoms as severe and did not require hospitalisation for any symptom. The disease was considered mild if the participants reported fewer than five symptoms as severe and did not require hospitalisation for any of them.

### Data analysis

All variables used in the study were categorical and were reported as frequency (percentages). The chi-square test was used to compare the difference in the sample characteristics by herbal medicine use status. Participants with missing data on herbal medicine use (yes, no) were excluded from the data analysis. Logistic regression analysis was performed to investigate the sociodemographic determinants of herbal medicine use. All analyses were run using Stata (Version 18, StataCorp, College Station, TX, USA). A *p*-value of < 0.05 (two-sided) was considered statistically significant.

## Results

### Population characteristics according to herbal medicine use

A total of 882 responses were received, with missing data in 31 responses regarding herbal medicine use (yes, no). A total of 851 responses were included in the analysis. Table 1 displays the sociodemographic characteristics of the study population according to the use of herbal medicines. Most of the study population were between 35 and 44 years of age (64.2%). Compared to non-users of herbal medicines, herbal medicines users were younger and had higher education levels. Notably, herbal medicines users had a higher number of positive COVID-19 tests and reported less severe symptoms compared to non-herbal medicines users; however, they also reported better overall health and required less medical attention and hospitalisation.

**Table 1.** Demographics of participants by herbal medicines use.

Herbal Medicines Use	No	Yes	*p*-Value
	N = 411	N = 440	
***Age (years)***			0.002
18–34	87 (21.2%)	98 (22.4%)	
35–54	250 (60.8%)	297 (68.0%)	
55+	74 (18.0%)	42 (9.6%)	
Missing	0	3	
***Sex***			<0.001
Male	241 (58.9%)	166 (38.5%)	
Female	168 (41.1%)	265 (61.5%)	
Missing	2	9	
***Nationality***			0.001
Qatari	58 (22.7%)	98 (35.3%)	
Non-Qatari	198 (77.3%)	180 (64.7%)	
Missing	155	162	
***Marital status***			0.605
Married	326 (79.7%)	360 (82.4%)	
Divorced/widowed	25 (6.1%)	24 (5.5%)	
Never married	58 (14.2%)	53 (12.1%)	
Missing	2	3	
***Education***			0.019
Below university	126 (31.1%)	94 (23.9%)	
University or above	279 (68.9%)	328 (76.1%)	
Missing	6	5	
***Employment***			0.036
No	35 (8.6%)	39 (9.2%)	
Yes	372 (91.4%)	385 (90.8%)	
Missing	4	16	
***Self-reported health***			0.047
Bad/very bad	7 (1.7%)	7 (1.6%)	
Fair	41 (10.0%)	24 (5.5%)	
Good/very good	357 (88.3%)	405 (92.9%)	
Missing	2	4	
***How many times did you test positive for COVID-19?***			<0.001
Never	24 (5.9%)	6 (1.4%)	
Once	275 (67.1%)	302 (68.9%)	
Two times	40 (9.8%)	71 (16.2%)	
Three times	38 (9.3%)	45 (10.3%)	
More than three times	33 (8.0%)	14 (3.2%)	
Missing	1	2	
***COVID severity***			0.002
Mild	307 (77.1%)	310 (71.4%)	
Moderate	34 (8.5%)	72 (16.6%)	
Severe	57 (14.3%)	52 (12.0%)	
Missing	13	7	
***Required medical attention***			0.668
No	263 (66.1%)	280 (64.7%)	
Yes	135 (33.9%)	153 (35.3%)	
Missing	13	7	
***Required hospitalisation***			0.318
No	341 (85.7%)	382 (88.0%)	
Yes	57 (14.3%)	52 (12.0%)	
Missing	13	6	

### Patterns and reasons for herbal medicine use by COVID-19 patients in Qatar

**Figure 1.**
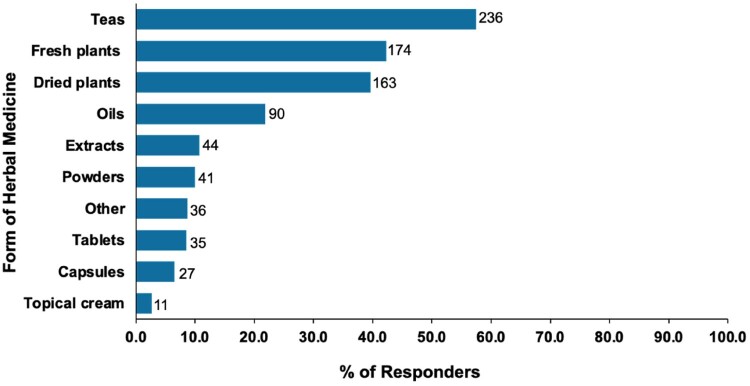
Forms of herbal remedies used by COVID-19 patients in Qatar. Bar labels denote the responses received for a herbal medicine form. The frequency of responses is reported as the number of responders divided by the number of respondents. Participants were allowed to choose multiple options when asked about the form of herbs they use.

Table 2 shows that the top three reported reasons for using herbal medicines were family tradition (42.0%), the belief that herbal medicines are more natural (34.8%), and the desire to improve health and survival (31.1%). The choice of herbal medicines was primarily based on personal preference and family traditions. Furthermore, the participants used herbal medicines in the form of teas, fresh plants, dried plants, oils, extracts, powders, tablets, capsules, or topical creams (Figure 1). Among the users of herbal medicines, teas (53.6%) were the preferred form, followed by fresh and dried plants (39.5% and 37%, respectively). Topical creams (2.5%) were the least common form of use (Figure 1).

**Table 2.** Reasons, and modes of herbal medicines use among COVID-19 patients in Qatar. Data are presented as n (%) for categorical measures.

	Total N = 440
***Reasons for Herbal Medicines Use***	
Family tradition	185 (42.0%)
It is more natural	153 (34.8%)
To improve your general health and ensure long term survival	137 (31.1%)
Culture/traditional medicine	128 (29.1%)
To prevent infection with COVID-19	116 (26.4%)
Belief in advantages of CAM practices	111 (25.2%)
To help in relaxation and feeling better psychologically	109 (24.8%)
To reduce the side effects/symptoms of conventional treatment	107 (24.3%)
To manage COVID-19 complications/progression	104 (23.6%)
To feel more in control over your health care	73 (16.6%)
To provide energy	54 (12.3%)
Internet/mass media	45 (10.2%)
Social media	40 (9.1%)
Feeling of having no alternative	37 (8.4%)
Friend	29 (6.6%)
Disappointment from conventional medical therapy	28 (6.4%)
Published studies in scientific journals	17 (3.9%)
Health care provider	6 (1.4%)
***How Herbal Medicines Were Chosen***	
Self-prescribed	181 (41.1%)
Family	181 (41.1%)
Friends	157 (35.7%)
Naturopath	46 (10.5%)
Herbalist	41 (9.3%)
Practitioner of traditional medicine	30 (6.8%)
Homeopath	22 (5.0%)

### Herbs used by COVID-19 patients in Qatar

**Figure 2.**
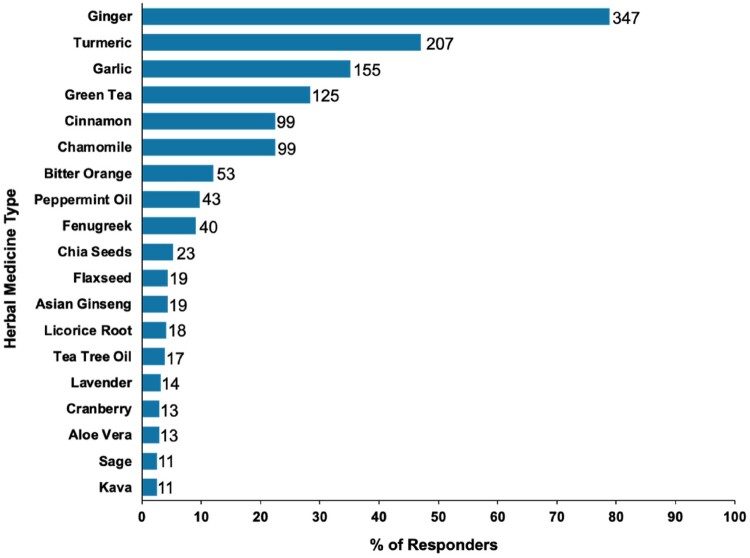
Frequency of use of herbal remedies by COVID-19 patients in Qatar. Bar labels denote the responses received for a herbal medicine type. The frequency of responses is reported as the number of responders divided by the number of respondents. Respondents were allowed to give multiple responses when asked about the herbs they use.

The frequency of use of the various herbs by the study respondents is shown in Figure 2. The most commonly used herbs were ginger, reported by 347 participants (78.9%), and turmeric by 207 participants (47.0%), followed by garlic (155 participants, 35.2%) and green tea (125 participants, 28.4%).

### Sociodemographic determinants of herbal medicines use by COVID-19 patients in Qatar

Table 3 presents the influence of sociodemographic characteristics, self-reported health status, and disease severity on the use of herbal medicines among COVID-19 patients. Overall, sex and nationality were shown to be the most significant drivers of the use of herbal medicines among COVID-19 patients. Notably, women were more likely to use herbal medicines compared to men (OR = 1.90, 95%CI: 1.30–2.77, *P* = 0.001). In addition, being a non-Qatari was associated with lower odds of using herbal medicines than being a Qatari (OR = 0.52, 95%CI: 0.33–0.80, *P* = 0.003). Age, marital status, education status, and employment status showed no significant association with the use of herbal medicines (Table 3). Furthermore, Participants who rated their health as fair were significantly less likely to use herbal medicines compared to those reporting good or very good health (OR = 0.50, 95% CI: 0.26–0.97, *P* = 0.041). While those with moderate COVID-19 symptoms appeared more likely to use herbal medicines than those with mild symptoms, the association was not statistically significant (OR = 1.70, 95% CI: 0.93–3.12, *P* = 0.081). No difference was observed among those with severe symptoms (OR = 0.99, 95% CI: 0.57–1.72, *P* = 0.992), indicating no clear trend in herbal medicine use across severity levels.

**Table 3.** Determinants of herbal medicines for COVID-19.

Herbal Medicines Use	OR	CI (95%)	*P*-value
***Age***			
18–34	1.00		
35–54	0.96	(0.60–1.52)	0.871
55+	0.56	(0.28–1.11)	0.098
***Sex***			
Male	1.00		
Female	1.90	(1.33–2.77)	0.001
***Nationality***			
Qatari	1.00		
Non-Qatari	0.52	(0.33–0.80)	0.003
***Marital Status***			
Married	1.00		
Never Married	0.73	(0.42–1.25)	0.258
Divorced/Widowed	0.71	(0.32–1.57)	0.405
***Education Status***			
Below University	1.00		
University or above	1.53	(0.98–2.39)	0.057
***Employment status***			
Unemployed	1.00		
Employed	0.94	(0.48–1.84)	0.867
***Severity***			
Mild	1.00		
Moderate	1.70	(0.93–3.12)	0.081
Severe	0.99	(0.57–1.72)	0.992
***Self-reported health***			
Good/very good	1.00		
Fair	0.50	(0.26–0.97)	0.041
Bad/very bad	1.09	(0.17–7.01)	0.098

### COVID-19 patients in Qatar self-reported benefits of herbal medicines use

Of the 440 herbal medicine users, 379 (88.6%) reported benefit while 42 (9.8%) reported that they were not sure, and 7 (1.6%) reported no benefit (Table 4). There was missing data from 12 users. The most commonly self-reported benefits of herbal medicine use, reported by the majority of the users, were for symptoms of sore throat (60.0%), and cough (55.0%) (Table 4).

**Table 4.** Self-reported benefits of herbal medicine use for COVID-19. Data are presented as n (%) for categorical measures.

COVID-19 patients who use herbal medicines	Total N = 440
***Did you benefit from herbal medicine use?***	
Yes	379 (86.1%)
No	7 (1.6%)
Not sure	42 (9.6%)
Missing	12
***Self-reported benefits of herbal medicine use***	
Fever ≥38°C	127 (28.9%)
Chills	65 (14.8%)
Fatigue	65 (14.8%)
Muscle ache (myalgia)	93 (21.1%)
Sore throat	264 (60.0%)
Cough	242 (55.0%)
Runny nose (rhinorrhea)	111 (25.2%)
Shortness of breath (dyspnoea)	51 (11.6%)
Wheezing	19 (4.3%)
Chest pain	28 (6.4%)
Other respiratory symptoms	31 (7.0%)
Headache	113 (25.7%)
Nausea/vomiting	32 (7.3%)
Abdominal pain	61 (13.9%)
Diarrhea	51 (11.6%)
Loss of sense of smell	54 (12.3%)
Loss of sense of taste	51 (11.6%)
Other	37 (8.4%)

## Discussion

### Summary of key findings

This study successfully reports the prevalence, reasons, uses, types, self-reported benefits, and sociodemographic determinants of herbal medicine use among COVID-19 patients in Qatar.

Over half (51.7%) of the 851 included participants reported using herbal medicine during their illness, suggesting a high prevalence within this population. Over half (51.7%) of the 851 participants reported using herbal medicine during their illness, indicating a high level of use within this population. Herbal medicine use was significantly higher among women and Qatari nationals. Younger and more educated individuals also tended to use herbal medicines more, though these trends were not statistically significant.

The most commonly used herbal remedies were ginger (78.9%), turmeric (47%), and garlic (35.2%); they were most frequently consumed as tea (53.6%), fresh plants (39.5%), and dried plants (37%). Notably, there was very low involvement of healthcare professionals (1.4%) in recommending herbal medicine; their use was most frequently self-prescribed (41.1%) or recommended by family (41.1%) and friends (35.7%).

The primary motivations for use were rooted in family tradition (42%), beliefs in natural remedies (34.8%), and desires to improve health or survival (31.1%). A large majority (88.6%) reported benefits, particularly relief from sore throat (60%) and cough (55%).

Interestingly, herbal medicine users reported milder symptoms and lower hospitalisation rates However, it is important to interpret this finding cautiously, as severity was based on self-reported hospitalisation which has recognised limitations as a reliable indicator of COVID-19 severity in Qatar (Sukik et al., 2025).

### Strengths and limitations

This investigation represents one of the first to explore herbal medicine use among COVID-19 patients within the Gulf Cooperation Council (GCC) region. It goes beyond simple prevalence reporting by examining a broad spectrum of related factors, which offers valuable insight for healthcare providers, policymakers, and public health educators aiming to guide safe and effective herbal medicine use during health emergencies.

The large sample size is a key strength of this study, offering adequate statistical power to detect associations and reliably estimate prevalence. Further, the statistical analysis identified independent predictors of herbal medicine use while controlling for potential confounding variables.

Conversely, the cross-sectional design of this study limits the ability to establish causality between herbal medicine use and reported outcomes (e.g. symptom improvement or reduced hospitalisation). The reliance on self-reported data introduces possible recall bias, especially concerning herbal medicine types and benefits. Additionally, recruitment via SMS may have biased the sample toward digitally literate individuals, underrepresenting older adults or those with limited digital literacy, potentially affecting generalisability. Moreover, the study did not evaluate the formulation quality, standardisation, or dosing of herbal products used, which may influence safety and effectiveness. Similarly, herbal medicine use and COVID-19 symptom severity were not clinically verified.

Unlike previous studies (Aydın Aksoy et al., 2024), this study did not assess household or family-level practices but focused only on individual-level use of herbal medicine. As a result, it may not have fully captured how caregiving roles and shared decision-making within households influence the use of herbal remedies during illness. Future research could explore these dimensions to better understand the dynamics of herbal medicine use in culturally specific family contexts.

Finally, COVID-19 vaccination status was not assessed in this study. Given that vaccination may influence symptom severity and hospitalisation risk, the absence of this variable limits our ability to account for its potential confounding effect when interpreting associations between herbal medicine use and COVID-19 outcomes.

Future studies should use longitudinal designs, verify symptoms and herbal product details clinically, and examine household practices. Despite limitations, this study offers valuable insights into herbal medicine use during COVID-19 in the GCC region.

### Interpretation of key findings

This study found a high prevalence of herbal medicine use during the COVID-19 pandemic in Qatar, consistent with global reports of CAM use ranging from 30% to 70% (Demeke et al., 2021). These findings mirror trends in the GCC and neighbouring countries – for example, usage rates of 22.1% in Saudi Arabia (Alyami et al., 2020), 54% in Turkey (Büşra Erarslan & Kültür, 2021), and 49% in Iran (Dehghan et al., 2022). Notably, an even higher percentage (93%) of use of natural or herbal products for COVID-19 prophylaxis was observed in an online survey in Saudi Arabia (Alotiby & Al-Harbi, 2021). In China, over 85% of patients received Traditional Chinese Medicine (TCM) in regions where traditional herbal remedies were officially integrated into COVID-19; as an adjunct treatment (Xing & Liu, 2021). This reinforces the notion that reliance on herbal remedies during the pandemic was a global phenomenon, not limited to any single region or culture.

The findings are consistent with local studies reporting widespread CAM and herbal use across various populations in Qatar. For example, 53% of patients with type 2 diabetes patients used CAM, mainly herbal powders (Mohamed et al., 2016), while high usage was noted among university students (Mamtani et al., 2015), and middle-aged women, particularly for nutritional and herbal therapies (Gerber et al., 2014). Moreover, 83% of general practitioners believe that CAM should be practiced in Qatar (Al Shaar et al., 2010). Regional studies similarly report common use among patients with chronic illnesses (Abuelgasim et al., 2018; AlBraik et al., 2008; Alnaimat et al., 2023; Kebede et al., 2021; Radwan et al., 2020). This high prevalence signals the need to further integrate CAM into public health considerations during pandemics and for authorities to develop and offer culturally relevant education on safe herbal use to counter misinformation and unmonitored self-treatment. These trends point to a high prevalence of CAM use in the region and signal the need to incorporate culturally appropriate education on safe herbal practices into public health strategies, particularly during pandemics.

Our study also identified demographic patterns in herbal use. Women were more likely than men to use herbal medicine, mirroring findings from other COVID-19 studies (Büşra Erarslan & Kültür, 2021; Dehghan et al., 2022), and earlier studies in the region (Abuelgasim et al., 2018; Alnaimat et al., 2023; Gerber et al., 2014). This is especially important in Middle Eastern settings where women often play a central role in family health decisions (Al Riyami et al., 2004; Yount, 2008), making them key targets for health messaging when considering tailoring of policy and education. Similarly, such tailoring of policy and education should also consider the cultural embeddedness of herbal medicines among Qatari nationals (Al Shaar et al., 2010; AlMusleh & Aboushanab, 2022), and the potentially higher use among educated youth. Such trends indicate that CAM engagement strategies should not assume a narrow target audience.

The commonly used herbs – ginger, turmeric, garlic and green tea, follow global trends observed during the pandemic. These herbs were among the most commonly used during COVID-19 pandemic due to their reported anti-inflammatory and immune-boosting properties (Ang et al., 2020, 2022a, 2022b; Panyod et al., 2020). The use of these herbs also aligns with recently reported ethnobotanical trends in Qatar and the region (El Alaa et al., 2025). Public health messages should acknowledge widely used local remedies and clearly address their safety, proper use, and potential interactions with conventional treatments. In parallel, pharmacovigilance systems should be adapted to monitor high-use herbs for side effects or interactions. Healthcare providers also need greater awareness of commonly used herbs and preparation methods – such as herbal teas – which, while seemingly harmless, may carry pharmacological activity.

One notable insight from our study is the limited involvement of physicians or pharmacists in guiding herbal medicine use. This study found that herbal medicine use was primarily self-directed or influenced by family and friends, rather than medical professionals, a trend that is echoed globally. In China, social media or relatives were the first information source about COVID-19 herbal treatments for most university students (Alotiby & Al-Harbi, 2021; Li et al., 2022; Wahab et al., 2023). Similarly, 66.7% of people using herbal supplements for COVID-19 prevention obtained information from the internet or social networks in Malaysia (Wahab et al., 2023), and word-of-mouth or online channels rather than medical consultation were the sources of information on herbal medicine use with COVID-19 in Saudi Arabia. This finding also resonates with other local and regional studies, which have indicated that CAM use is largely driven by non-medical sources (Abouelela et al., 2025; Abuelgasim et al., 2018; Alotiby & Al-Harbi, 2021; Alyami et al., 2020). Al Shaar et al., in a study of general practitioners (GPs) in Qatar, noted that GPs, despite being aware of CAM, rarely actively discussed it with patients. This gap emphasises an opportunity for policy and clinical practice: educating providers to integrate CAM discussions into clinical consultations (Al Shaar et al., 2010). In fact, many patients may have hesitated to inform doctors of their herbal usage, fearing dismissal, or did not view it as necessary to disclose since these products are easily accessible over-the-counter or from other sources (online, herbalists, etc.). On the provider side, pandemic-related pressures may have deprioritised asking about herbal or supplement use. The outcome is that much of the population was essentially self-medicating with no professional input. Such a scenario can be problematic: without medical guidance, patients risk potential herb-drug interactions, use of inappropriate remedies, or excessive dosing.

Herbal interventions hold the potential to improve patient safety and offer an opportunity to further engage with the cultural beliefs of patients and address misconceptions regarding the safe use of CAMs. For example, the perceived naturalness, historical and cultural familiarity, and immune-boosting beliefs that were common motivators for herbal medicine use reported in this study, in studies from the region (Alotiby & Al-Harbi, 2021; Alyami et al., 2020; Dehghan et al., 2022), and internationally (Ang et al., 2020, 2022a, 2022b; Li et al., 2022; Panyod et al., 2020; Soltani et al., 2023; Wahab et al., 2023), likely reflect deeply rooted cultural and emotional drivers which will require additional sensitivity for healthcare professionals to explore.

Our finding that herbal medicine users reported fewer symptoms and lower hospitalisation rates resonates with the findings of randomised controlled trials; it should be noted that these studies investigated herbal medicine usage alongside conventional treatments. Most community-based studies could not establish a causal link between herbal medicine and improved clinical outcomes in COVID-19 patients (Demeke et al., 2021; Panyod et al., 2020; Yang et al., 2020). Further research is required to determine whether herbal medicine plays a role in health outcomes or whether users are less severely ill to begin with. This finding reinforces the importance of not positioning herbal medicine as a substitute for evidence-based COVID-19 treatments but potentially as a complementary modality under guidance.

In summary, herbal medicine use was common during COVID-19 in the GCC, with higher usage among women and certain cultural groups. Many participants reported positive effects, although most used herbs without professional medical advice. This points to a gap between patients and healthcare providers that needs addressing. Future studies should focus on confirming the safety and benefits of these remedies and understanding cultural influences on their use. Overall, integrating herbal medicine discussions into clinical care could improve patient outcomes and safety.

## Conclusion

This study found that more than half of individuals with confirmed COVID-19 in Qatar used herbal remedies, primarily ginger, turmeric, garlic, and green tea, to manage symptoms and support immunity. Use was more common among women and Qatari nationals and was largely self-directed or influenced by family and social media, with minimal input from healthcare providers. These findings reflect strong cultural familiarity with herbal products and align with global patterns of complementary medicine use during the pandemic. While participants reported symptom relief and milder illness, the cross-sectional design and self-reported data limit causal interpretation. Future research should assess herbal medicine safety and efficacy and explore strategies to integrate culturally appropriate, evidence-informed guidance into public health and clinical practice.

## Supplementary Material

Supplemental Material
