# Supplementary material for: Prevalence, patterns, drivers, and perceived benefits of herbal medicine use in COVID-19 patients in Qatar
Source: J Pharm Policy Pract. 2025 Jul 23;18(1):2533258. doi: 10.1080/20523211.2025.2533258 (PMC12288189; doi:10.1080/20523211.2025.2533258)
Supplement: Supplemental Material [file JPPP_A_2533258_SM8253.pdf]

<u>**Survey**</u>

Use, types, and self-reported harms and benefits of herbal medicines in Primary Health Care Corporation registered population with previous history of confirmed COVID-19

Dear PHCC client,
This SMS is to invite you to participate in a survey that aims to understand the use of herbal medicines with COVID-19.
Your participation is important to help understand the extent of use of herbal medicines and their self-reported harms/benefits, especially in COVID-19. Completing the questionnaire should take you no more than 10 minutes. There is no expected harm or discomfort resulting from participating in this study. Likewise, there is no direct benefit to the participants, but the study results may help to provide new information on herbal medicine use in Qatar. Participation in this study is voluntary and anonymous. You may withdraw and discontinue participation at any time without affecting you. However, we will not be able to delete your response due to the anonymous nature of the study. All information and records generated in this study are strictly confidential.
If you have any questions or concerns about this research, please contact:
Research Supervisor: Dr. Abdullah Shaito, Qatar University.
Email: abdshaito@qu.edu.qa
Tel: 44035680

Your contribution is highly appreciated.
Sincerely,

<u>**Section A: Information about you**</u>

1- How old are you?

- ☐ 18 – 24
- ☐ 25 - 34
- ☐ 35 - 44
- ☐ 45 - 54
- ☐ 55 - 64
- ☐ > 65

2- What is your gender?

- ☐ Man
- ☐ Woman

3- What is your nationality?

____

4- What is your highest level of education?

- ☐ Never attended school
- ☐ Primary school (Class 1 to 6)
- ☐ Secondary school (Class 7 to 12)
- ☐ Trade/technical/vocational qualification
- ☐ Diploma/Bachelor's degree
- ☐ Post graduate degre

Approved Date: November 10, 2022
Qatar University Institutional Review Board (QU-IRB)

Approved by PHCC Department of Cllinical Research - PHCC/DCR/2022/06/040

5- What is your current employment status?

- ☐ Employed for a salary
- ☐ Self-employed
- ☐ Unemployed less than 1 year
- ☐ Unemployed 1 year or more
- ☐ Student
- ☐ Retired

6- What is your marital status?

- ☐ Never married before
- ☐ Married
- ☐ Separated
- ☐ Divorced
- ☐ Widowed

Section B: Information about your health status

7- How is your health in general? Would you say it is

- ☐ Very good
- ☐ Good
- ☐ Fair
- ☐ Bad
- ☐ Very bad

8- Do you have any physical or mental health conditions or illnesses lasting or expected to last for 12 months or more?

- ☐ Yes
- ☐ No

If yes, please record all conditions or illnesses (you can choose more than one option):

- ☐ Cancer
- ☐ Hypertension
- ☐ Coronary artery disease
- ☐ Congestive heart failure
- ☐ Asthma
- ☐ Chronic obstructive pulmonary disease
- ☐ Obstructive sleep apnea
- ☐ History of solid organ transplant
- ☐ Chronic kidney disease
- ☐ End-staged kidney disease
- ☐ Cirrhosis
- ☐ Chronic liver disease
- ☐ Hepatitis B
- ☐ Hepatitis C
- ☐ Diabetes

9- Do any of your conditions or illnesses affect you in any of the following areas (you can choose more than one option):?

- ☐ Vision (e.g. blindness or partial sight)
- ☐ Hearing (e.g. deafness or partial hearing)
- ☐ Mobility (e.g. walking short distances or climbing stairs)
- ☐ Dexterity (e.g. lifting and carrying objects, using a keyboard)
- ☐ Learning or understanding or concentrating
- ☐ Memory
- ☐ Mental health
- ☐ Stamina or breathing or fatigue
- ☐ Socially or behaviourally (e.g. associated with autism, Attention Deficit Disorder or Asperger's syndrome)
- ☐ Other (PLEASE SPECIFY)______________________

<u>Section C: Information about your COVID-19 infection</u>

10- How many times did you test positive for COVID-19?

- ☐ Never
- ☐ Once
- ☐ Twice
- ☐ Three times
- ☐ More than three times

11- When was the last time you tested positive?

- ☐ 3 months ago
- ☐ 4-6 months ago
- ☐ 6- 12 months ago
- ☐ More than 12 months ago

12- Which of the following COVID-19 symptoms did you experience (you can choose more than one option):?

Symptoms	Severity
Fever ≥38°C	☐ Mild<br>☐ Moderate<br>☐ Severe
Chills	☐ Mild<br>☐ Moderate<br>☐ Severe
Fatigue	☐ Mild<br>☐ Moderate<br>☐ Severe
Muscle ache (myalgia)	☐ Mild<br>☐ Moderate<br>☐ Severe
Sore throat	☐ Mild<br>☐ Moderate<br>☐ Severe
Cough	☐ Mild<br>☐ Moderate<br>☐ Severe
Runny nose (rhinorrea)	☐ Mild<br>☐ Moderate<br>☐ Severe
Shortness of breath (dyspnea)	☐ Mild<br>☐ Moderate<br>☐ Severe
Wheezing	☐ Mild<br>☐ Moderate<br>☐ Severe
Chest pain	☐ Mild<br>☐ Moderate

Approved Date: November 10, 2022

Qatar University Institutional Review Board (QU-IRB)

Approved by PHCC Department of Cllinical Research - PHCC/DCR/2022/06/040

	☐ Severe
Other respiratory symptoms	☐ Mild<br>☐ Moderate<br>☐ Severe
Headache	☐ Mild<br>☐ Moderate<br>☐ Severe
Nausea/vomiting	☐ Mild<br>☐ Moderate<br>☐ Severe
Abdominal pain	☐ Mild<br>☐ Moderate<br>☐ Severe
Diarrhoea	☐ Mild<br>☐ Moderate<br>☐ Severe
Loss of sense of smell	☐ Mild<br>☐ Moderate<br>☐ Severe
Loss of sense of taste	☐ Mild<br>☐ Moderate<br>☐ Severe

13- Did any of your symptoms require you to seek medical attention?

- ☐ Yes
- ☐ No

14- Did any of your symptoms require you to be hospitalized?

- ☐ Yes
- ☐ No

Section D: Use and types of herbal medicines

15- Have you used herbal medicines for any reason**, other than COVID-19**?

- ☐ Yes, rarely
- ☐ Yes, sometimes
- ☐ Yes, often
- ☐ No

16- Have you used herbal medicines for **COVID-19**?

- ☐ Yes
- ☐ No

17- If you have not used herbal medicines, why not? (you can choose more than one option)

- ☐ I never heard of it
- ☐ I'm afraid of the side effects
- ☐ I don't believe in it
- ☐ The doctor didn't prescribe it
- ☐ Not to have additional burden
- ☐ Other, please specify ____________

18- If you have used herbal medicines for **COVID-19,** what form were they in? (you can choose more than one option):

- ☐ Tablets
- ☐ Capsules
- ☐ Topical cream
- ☐ Powders
- ☐ Teas
- ☐ Extracts
- ☐ Fresh plants
- ☐ Dried plants
- ☐ Oils
- ☐ Other, specify ____________

19- If you have used herbal medicines for **COVID-19,** what key ingredient did they include? (you can choose more than one option)

- Acai
- Aloe Vera
- Asian Ginseng
- Astragalus
- Bilberry
- Bitter Orange
- Black Cohosh
- Butterbur
- Cat's Claw
- Chamomile
- Chasteberry
- Chia Seeds
- Cinnamon
- Cranberry
- Dandelion
- Echinacea
- Echinacea
- Elderberry
- Ephedra
- European Mistletoe
- Evening Primrose Oil
- Fenugreek
- Feverfew
- Flaxseed
- Garlic
- Ginger
- Gingko
- Goldenseal
- Grape Seed Extract
- Green Tea
- Hawthorn
- Hoodia
- Horse Chestnut
- Kava
- Lavender
- Licorice Root
- Milk Thistle
- Mugwort
- Noni
- Passionflower
- Peppermint Oil
- Red Clover

Approved Date: November 10, 2022
Qatar University Institutional Review Board (QU-IRB)

Approved by PHCC Department of Cllinical Research - PHCC/DCR/2022/06/040

- Red Yeast Rice
- Sage
- Saw Palmetto
- Soy
- St. John's Wort
- Tea Tree Oil
- Thunder God Vine
- Turmeric
- Valerian
- Yohimbe
- Other? Specify__________

20- Who recommended you to use herbal medicines for COVID-19? (you can choose more than one option)

- ☐ Self-prescribed
- ☐ Friends
- ☐ Allopathy doctor
- ☐ Massage therapist
- ☐ Acupuncturist
- ☐ Practitioner of traditional medicine
- ☐ Naturopath
- ☐ Homeopath
- ☐ Herbalist
- ☐ Other specify ________

21- Why did you use herbal medicine for COVID-19? (you can choose more than one option)

- To prevent infection with COVID-19
- To manage COVID-19 complications/progression
- To reduce the side effects/symptoms of conventional treatment
- To help in relaxation and feeling better psychologically
- To improve your general health and ensure long term survival
- To feel more in control over your health care
- To provide energy
- Disappointment from conventional medical therapy
- Feeling of having no alternative
- Belief in advantages of CAM practices
- Family tradition
- Friend
- Culture/ Traditional Medicine
- It is more natural
- Curiosity
- Social media
- Published Studies in Scientific Journal
- Health care provider
- Internet (Youtube, Google) Mass Media
- Other, please specify: _____________

22- Did you use conventional (modern) medicines together with herbal medicine for COVID-19?

- ☐ Yes
- ☐ No

23- Where from did you purchase/get your herbal CAM? (you can choose more than one option)

- ☐ Local supermarket.
- ☐ Alternative medicine practitioner
- ☐ Shopping online.
- ☐ Relative or friend.
- ☐ Other, specify _________

24- How much did you spend on herbal medicines to treat COVID-19?

- < QAR 100
- QAR 100 – 250
- QAR 250 - 500
- > QAR 250 - 500

Section E: Benefits and harms of herbal medicines

25- Did you benefit from using herbal medicines for COVID-19?

- ☐ Yes
- ☐ No
- ☐ Not sure

If yes, what benefits did you experience? (you can choose more than one option)
Reduction/ improvement in:

- Fever ≥38°C
- Chills
- Fatigue
- Muscle ache (myalgia)
- Sore throat
- Cough
- Runny nose (rhinorrea)
- Shortness of breath (dyspnea)
- Wheezing
- Chest pain
- Other respiratory symptoms
- Headache
- Nausea/vomiting
- Abdominal pain
- Diarrhoea
- Loss of sense of smell
- Loss of sense of taste
- Other

Thank you very much for your participation
